# A reliable radiographic measurement for evaluation of normal distal tibiofibular syndesmosis: a multi-detector computed tomography study in adults

**DOI:** 10.1186/s13047-015-0093-6

**Published:** 2015-07-25

**Authors:** Yanxi Chen, Minfei Qiang, Kun Zhang, Haobo Li, Hao Dai

**Affiliations:** Department of Orthopedic Trauma, Shanghai East Hospital, Tongji University School of Medicine, 150 Jimo Road, 200120 Shanghai, China

**Keywords:** Ankle joint, Tomography, Spiral computed, Imaging, Three-dimensional, Image processing, Computer-assisted, Anatomy

## Abstract

**Background:**

Syndesmotic injury may be difficult to diagnose, and radiological evaluation is very important. The purpose of this study was to offer a series of reliable and repeatable normal tibiofibular syndesmosis parameters in diagnosing injuries of the syndesmosis.

**Methods:**

Multi-detector computed tomography (MDCT) and radiographs of the distal tibiofibular syndesmosis in 484 cases were retrospectively reviewed. Relevant parameters included the tibiofibular clear space (TCS), the tibiofibular overlap (TFO), the depth of the incisura fibularis (IFD), and the height of the incisura fibularis (IFH), which were measured by novel three-dimensional (3-D) and two-dimensional (2-D) techniques. The distance between the measuring plane of the distal tibiofibular syndesmosis and the tibial plafond was measured. Intra- and inter-rater reliability was assessed by intraclass correlation coefficient (ICC) and the root mean square standard deviation (RMS-SD), to determine measurement precision. Sex differences of parameters were analyzed using analysis of covariance (ANCOVA) with body height as the covariate. Paired sample *t*-testing was used to compare parameters in different image modalities, including radiography, and 2-D and 3-D CT.

**Results:**

The reliability of the 3-D images measurement (ICC range, 0.907 to 0.972) was greater than that for the 2-D axial images (ICC range, 0.895 to 0.927), and the AP view radiographs (ICC range, 0.742 to 0.838). The intra-rater RMS-SD of the 3-D CT, 2-D CT and radiographic measurements were less than 0.94 mm, 0.26 mm, and 2.87 mm, respectively. The measuring plane of the distal tibiofibular syndesmosis showed the sex difference, which was 12.1 mm proximal to the tibial plafond in the male group and 7.8 mm in the female group. In this plane, the parameters for tibiofibular syndesmosis were measured in different image modalities. All variables were significantly different between females and males (*p* < 0.05).

**Conclusions:**

3-D measurement technique could be helpful to identify the precise measurement planes for syndesmosis, which were not at the fixed level above the tibial plafond because of the sex difference. In this plane, reliable measurement results could be provided, in either 2-D or 3-D MDCT images.

**Electronic supplementary material:**

The online version of this article (doi:10.1186/s13047-015-0093-6) contains supplementary material, which is available to authorized users.

## Background

It is estimated that 13 % of all ankle fractures [1], and 20 % of operatively treated ankle fractures are accompanied by a syndesmotic injury [2, 3]. Cedell documented the incidence of syndesmotic sprains as only 1 to 10 % of all ankle ligament injuries [4]. Others believe that the incidence may be as high as 40 % in athletes [5]. Several clinical tests that are most commonly used in diagnosing injuries of the syndesmosis are described [5–8]. However, none of these tests has a high predictive value for acute disruption of the syndesmosis [9]. Therefore, radiological evaluation of tibiofibular syndesmosis injury is very important, with attention to the tibiofibular clear space (TCS) and tibiofibular overlap (TFO) [10]. Syndesmotic injury may be difficult to diagnose using radiographs. In the past, the anatomical measurement of the distal tibiofibular syndesmosis was based on a two-dimensional (2-D) plane. The main methods included cadaver study [10–14], radiographs [10, 11, 15, 16], and axial computed tomography (CT) scan images [12, 13, 17–19]. Reviewing the above studies, first, it is hard to obtain cadaver specimens, and difficult to carry out a prospective study in groups with large samples. Second, radiographs may be limited by the position of the patient’s ankle and the angle of the beam, which may not be completely controlled, especially when post-traumatic [20]. Third, the selection of axial CT scan images is also affected by the position of the ankle and the reconstruction interval. Recent studies have evaluated CT images to define the normal appearance of the syndesmosis [17, 18, 21], but no clear consensus has been reached.

In the present study, we proposed (1) to present novel techniques to measure the normal distal tibiofibular syndesmosis based on multi-detector CT (MDCT); (2) to evaluate the reliability of these measurement techniques in three imaging modalities including plain film radiography, 2-D axial CT images and 3-D CT images, using the intraclass correlation coefficient (ICC) and the root mean square standard deviation (RMS-SD) to determine measurement precision; (3) to determine whether there was a difference in the measurements among different imaging modalities using comparative statistics. If validated, these standardized measurements would provide a series of reliable and repeatable parameters in male and female groups respectively and serve as normal control data for injuries of the tibiofibular syndesmosis.

## Methods

### Study participants

From January 2009 to December 2013, 484 participants were retrospectively included in the study. The most common presenting diagnoses among included participants were metatarsal/phalangeal fractures, hallux valgus and plantar fasciitis, while people diagnosed by radiographs with ankle deformities, ankle fractures, ankle osteoarthritis, ankle tumors and other ankle diseases were not selected. All participants underwent a complete history and detailed musculoskeletal physical examination by a fellowship-trained foot and ankle surgeon. These participants had normal ankle radiographs. The research protocol (Ethics Approval No: #2013020) was approved by the institutional review committee of East Hospital of Tongji University in Shanghai, China. Written informed consents were obtained.

### Radiology technique

The anteroposterior (AP) radiographs of the ankle in a non-weight-bearing position were obtained by experienced technicians working in a foot and ankle specialty clinic. CT scans were performed by a 16–detector CT scanner (GE Light-Speed CT; Waukesha, WI, USA). Imaging parameters for plain scanning images of the normal distal tibiofibular syndesmosis were as follows: section thickness, 0.625 mm; tube voltage, 120 kVp; pitch, 1.375; matrix, 512 × 512. All the data were reviewed retrospectively by a musculoskeletal radiologist and a foot and ankle surgeon, which were saved as DICOM 3.0 format (.dcm).

### Image post-processing

**Fig. 1:**
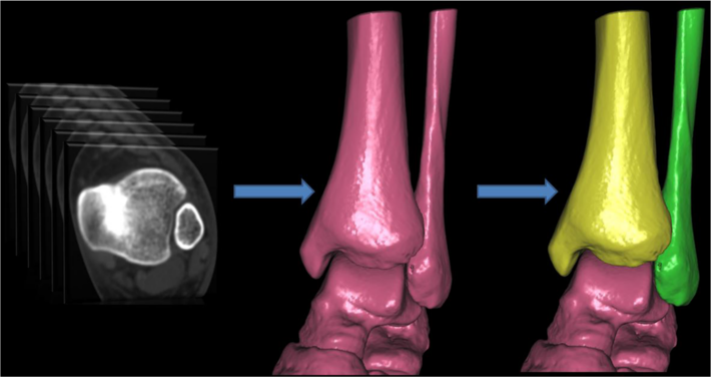
3-D images of the ankle were extracted by 3-D interactive and automatic segmentation technique after SSD reconstruction. After the tibia and fibula had been selected in the software, they gradually turned yellow and green, respectively. The other bones including the talus were still red

The thin-slice CT axial images of all research subjects were input into the computer -aided orthopedic clinical research system (SuperImage orthopedics edition 1.1, Cybermed Ltd, Shanghai, China) [22–24]. The software was developed by Java language on NetBeans (Sun Microsystems, Inc., Santa Clara, CA, USA) and OpenInventor (Mercury Computer Systems/TGS Unit, San Diego, CA, USA) platforms. In this system, the 3-D images of the ankle joint were reconstructed by a surface shaded display (SSD) algorithm with a reconstruction interval of 0.625 mm. The density threshold was 150 HU, and automatic removal of the image size was less than 500 mm^3^ [25–28]. The 3-D interactive and automatic segmentation technique was applied, so the operator could distinguish the bones in the 3-D SSD image. Different colors were assigned to different bones (Fig. 1).

### Measurement methods

Distal tibiofibular syndesmosis related morphological parameters were measured according to combined 3-D measurement techniques including essential elements of point, line and surface in the 3-D CT images [22, 23, 25], and traditional measurement techniques in the plain film radiography and 2-D axial CT images. The major procedures for 3-D measurements are shown in the supplemental content [see Additional file 1]. All measurements were made with a virtual ruler on digital images obtained and rounded to the nearest 0.1 mm.

#### Measuring procedures in the 3-D image were as follows

1. According to the geometric algorithm that any developed curve surface is similar to a flat surface, hide the talus and click the distal tibia articular surface to create a pseudo-straighten plane as the tibial plafond (plane X) (Fig. 2a) [28].

**Fig. 2:**
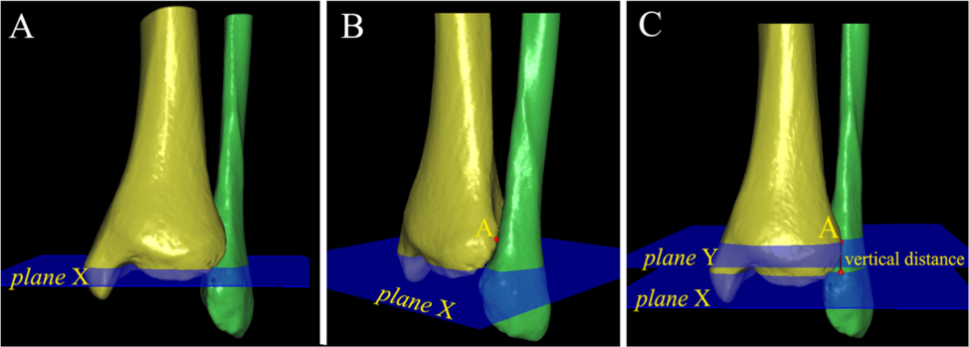
The measurement plane (plane Y) was selected individually. **a** The talus was hidden and the distal tibia articular surface was clicked to create a pseudo-straight plane as the tibial plafond (plane X). **b** “Point A” was selected on the anterior tubercle, which was defined as the intersection of the tangential along the anterior and posterior tubercle. **c** Selecting the measuring plane: the plane via point A paralleling the tibial plafond (plane X), called “plane Y”. The perpendicular distance from plane X to plane Y was measured
2. Point A on the anterior tubercle is defined as the intersection of the tangential along the anterior and posterior tibial tubercle (Fig. 2b).
3. Select the measuring plane: the plane via point A and parallel to the tibial plafond (plane X), which is called “plane Y” (Fig. 2c).
4. Anatomical landmarks in the plane Y are selected including point A, point B (the nearest point to the point A), point C on the posterior tubercle (the intersection of the tangential to the anterior and posterior tubercle), point D (the nearest point of the fibula to the posterior tubercle), and point E (the bottom point of incisura fibularis) (Figs. 3 and 4).

**Fig. 3:**
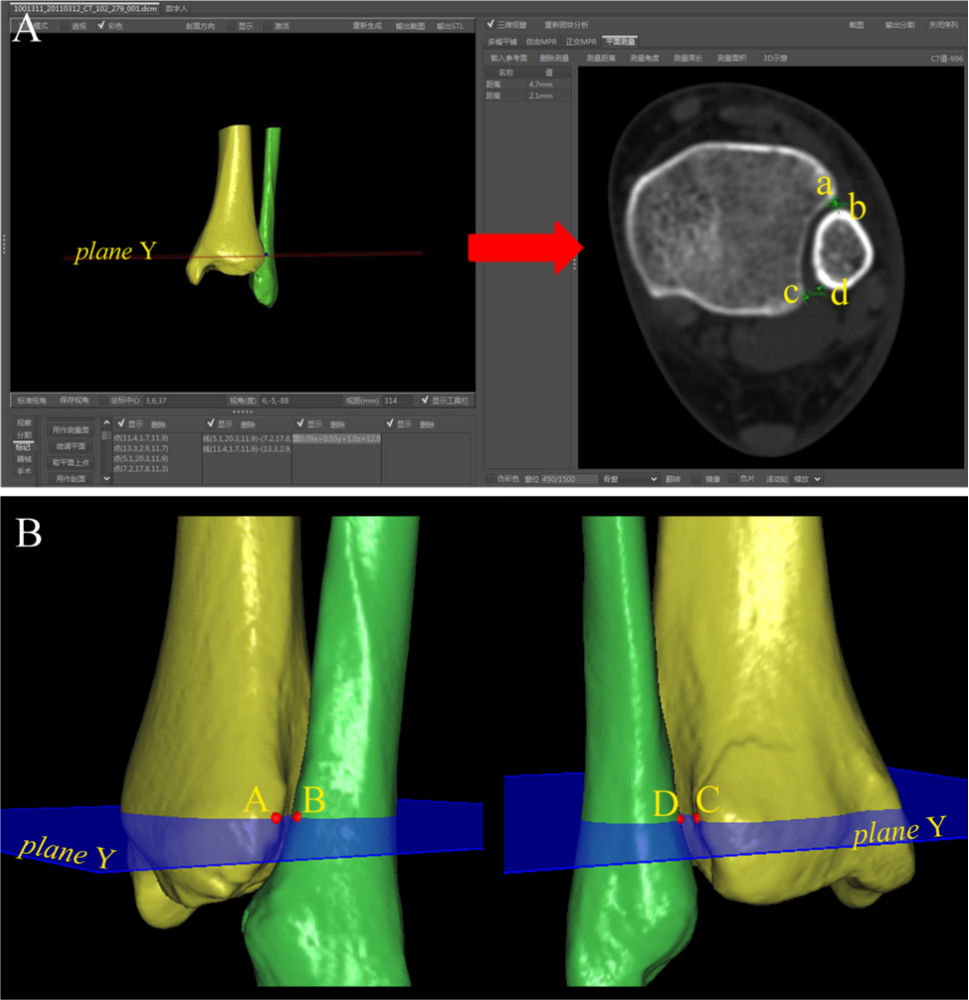
The tibiofibular clear space was measured in the 2-D axial and 3-D images of CT scans at same level of plane Y (red arrow). **a** TCS-A = anterior tibiofibular clear space (line ab); TCS-P = posterior tibiofibular clear space (line cd). **b** TCS-A (line AB) and TCS-P (line CD) in 3-D images were measured

**Fig. 4:**
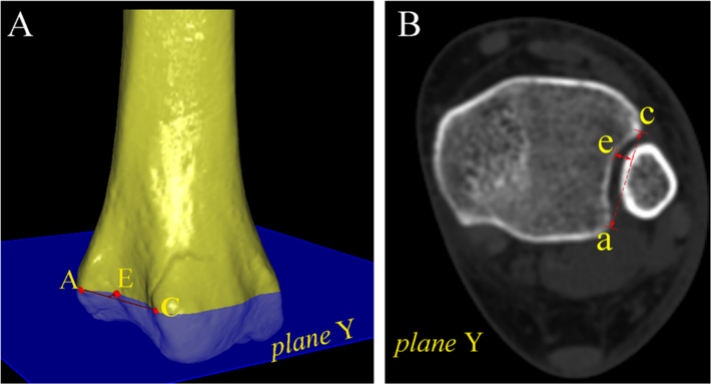
The depth of incisura fibularis in the **a** 3-D and **b** 2-D images were measured as the perpendicular distance from the bottom point of incisura fibularis (point E or point e) to line AC or line ac, respectively
5. Select the proximal tip of incisura fibularis as “point F” (Fig. 5).

**Fig. 5:**
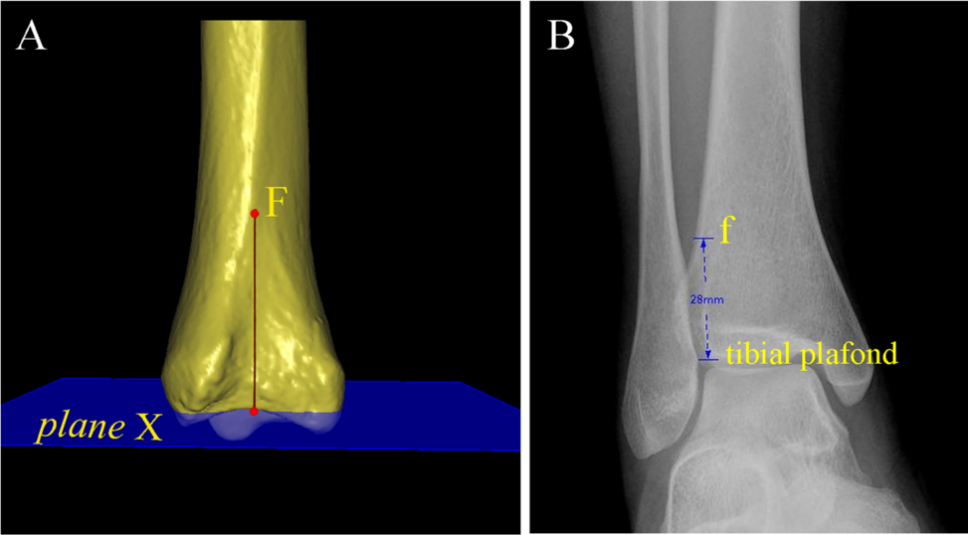
The measurement of the height of incisura fibularis was carried out. **a** The proximal tip of incisura fibularis was selected as “point F”, and the vertical distance from the point F to plane X in the 3-D image was measured. **b** The height of incisura fibularis in the AP view radiograph was described as the perpendicular distance from the proximal tip of incisura fibularis to the tibial plafond

#### Measurement parameters were as follows

1. The proximal distance between the measuring plane and the tibial plafond in the 3-D image measured the vertical distance from plane X to plane Y, abbreviated “XY” (Fig. 2).
2. The TCS divided into two parts, i.e., anterior and posterior, in the 3-D image and 2-D axial image of CT scans, anterior and posterior, abbreviated “TCS-A” and “TCS-P”. The measuring plane in the 3-D image was as a 2-D axial image selection plane, and measured the distance from point A to point B, and point C to point D in the 3-D and 2-D axial image (Fig. 3).
3. The TCS and TFO on the AP view radiographs measured the distance between the lateral border of the posterior tubercle and the medial border of the fibula, and between the medial border of the fibula and the lateral border of the anterior distal tibial tubercle, at the level of plane Y (Fig. 6).

**Fig. 6:**
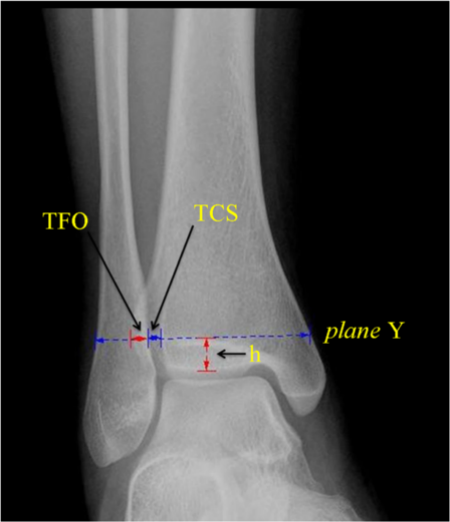
The TCS and TFO in the radiographs were measured at the level of plane Y. The “h” was not invariable, which was the perpendicular distance from plane Y (selected in the 3-D image) to the tibial plafond on the anteroposterior view. TCS = tibiofibular clear space; TFO = tibiofibular overlap
4. The depth of the incisura fibularis in the 3-D and 2-D images, abbreviated “IFD”, measured the vertical distance from point E to the line between point A and C in the 3-D and 2-D axial images (Fig. 4).
5. The height of the incisura fibularis in the 3-D image and the AP view radiographs, abbreviated “IFH”, measured the vertical distance from point F to plane X (tibial plafond) (Fig. 5).

### Intraclass correlation coefficient

The ICC was used for reliability to test at a desired lower limit of 0.8 and a 95 % confidence interval (CI) of 0.2. We randomly selected ankle radiographs and CT scans of 102 participants to assess the intra- and inter-rater reliability. The three foot and ankle surgeons with clinic experience of 14, 10 and 3 years performed the measurements independently. Before the initiation of the first measurement, one conference was held to uniformly educate all the authors on the measurement methods. No additional training sessions were performed among the three raters. The main examiner finished all the measurements of 484 subjects. The 102 subjects were measured again by the other examiners in different sessions. To determine intra-rater reliability, the main examiner repeated the measurements for 102 subjects with an interval of 4 weeks.

### Statistical analysis

The ICCs were used to assess the intra- and inter-rater reliability. Absolute agreement, as a two-way random model was selected and 95 % confidence interval (CI) was determined in the setting of a single measurement. Normal distributed measurement data were represented with mean ± standard deviation (SD), and statistical analysis methods included analysis of covariance (ANCOVA) with body height as the covariate for sex differences and paired sample *t*-testing for measurement technology differences. A statistical difference was defined as *p* value less than 0.05. The RMS-SD was calculated, and was used to assess the 3-D measuring precision of metatarsal and tarsal bones from quantitative CT and 3-D morphology of the calcaneus at the level of the individual participant [23, 29]. Intra- and inter-rater precision were calculated for all parameters as RMS-SD:

$$ RMS-SD=\sqrt{\frac{{\displaystyle \sum S{D}^2}}{N}} $$

The SD in the equation was calculated by repeat measures of each parameter for each of the 102 subjects. SD values in inter-rater precision represented variability across two raters. SD values in intra-rater precision represented variability after the repeated measurements by the same rater.

## Results

Four hundred and eighty-four participants included 219 women (mean age, 38.5 years; age, 20 to 55 years) and 265 men (mean age, 40.2 years; range, 21 to 55 years). The mean body height was 1.61 m (range, 1.55 to 1.70) in the female group and 1.73 m (range, 1.63 to 1.82) in the male group (*p* < 0.05).

The plane through point A and parallel to the tibial plafond was identified as a unique and precise measuring plane in 3-D CT, 2-D CT and plain film radiography. The mean proximal distance between the measuring plane and the tibial plafond in the 3-D image was 12.1 mm (range, 8.4 to 15.2) in the male group and 7.8 mm (range, 6.4 to 9.2) in the female group. In this plane, the parameters for tibiofibular syndesmosis were measured in different image modalities. All variables were significantly different between females and males after controlling for body height as the covariate (*p* < 0.05) (Table 1).

**Table 1:** Measurements of distal tibiofibular syndesmosis

Mean (range)	3-D SSD		2-D Axial		X ray	
	Male	Female	Male	Female	Male	Female
XY (mm)	12.1^*^ (8.4 to 15.2)	7.8^*^ (6.4 to 9.2)	NA	NA	NA	NA
TCS-A(mm)	2.8^*^ (2.2 to 3.5)	1.8^*^ (1.4 to 2.5)	2.8^*^ (2.3 to 3.7)	1.8^*^ (1.4 to 2.9)	NA	NA
TCS-P(mm)	3.6^*^ (2.9 to 4.3)	2.9^*^ (2.4 to 3.6)	3.7^*^ (2.6 to 5.2)	2.8^*^ (2.2 to 3.8)	NA	NA
TCS (mm)	NA	NA	NA	NA	4.1^*^ (2.1 to 5.8)	3.7^*^ (2.4 to 5.1)
TFO (mm)	NA	NA	NA	NA	5.5^*^ (3.1 to 8.2)	3.8^*^ (2.3 to 6.1)
IFD (mm)	5.1^*^ (4.0 to 6.3)	4.2^*^ (3.2 to 5.2)	5.0^*^ (3.6 to 6.5)	4.3^*^ (3.0 to 5.6)	NA	NA
IFH (mm)	35.1^*^ (28.6 to 39.8)	33.7^*^ (30.2 to 37.5)	NA	NA	28.3^*^ (18.4 to 37.2)	25.6^*^ (20.1 to 31.5)

^*^ *p* < 0.05 indicates a significant sex difference in parameters using ANCOVA with body height as the covariate; *NA* not applicable, *XY* vertical distance from plane X to plane Y, *TCS-A* anterior tibiofibular clear space, *TCS-P* posterior tibiofibular clear space, *TCS* tibiofibular clear space, *TFO* tibiofibular overlap, *IFD* depth of incisura fibularis, *IFH* height of incisura fibularis

With regard to parameters in different image modalities, neither TCS-A nor TCS-P was significantly different between 3-D and 2-D axial images in the same measurement plane (*p* > 0.05). According to the TCS definition [15], the TCS-P in the 3-D or 2-D CT images was equivalent to the TCS in the AP view radiographs, but the two parameters were significantly different (*p* < 0.05). The IFD was not significantly different between 3-D and 2-D images (*p* > 0.05). The IFH showed a significant difference between 3-D images and radiographs (*p* < 0.05) (Table 2).

**Table 2:** Comparison of parameters in different image modalities

	*t* value	*p* value^*^
3-D CT/2D CT		
TCS-A	1.870	0.062
TCS-P	0.061	0.951
IFD	−1.758	0.079
3-D CT/Radiographic		
TCS-P/TCS	−19.878	<0.001
IFH	68.835	< 0.001
2-D CT/Radiographic		
TCS-P/TCS	−22.067	< 0.001

^*^Paired *t*-tests; *TCS-A* anterior tibiofibular clear space, *TCS-P* posterior tibiofibular clear space, *IFD* depth of incisura fibularis, *TCS* tibiofibular clear space, *IFH* height of incisura fibularis

The intra- and inter-rater reliability of all variables showed the ICC range from 0.742 to 0.972 (Table 3). The reliability of the 3-D images measurement (ICC range, 0.907 to 0.972) was greater than the reliability of the 2-D axial images (ICC range, 0.895 to 0.927) and the AP view radiographs (ICC range, 0.742 to 0.838). Three radiography reviewers attributed the lower reliability to the difficulty in precisely measuring the IFH with radiography. The RMS-SD for intra- and inter-rater precision of the 3-D and 2-D axial image and radiographic measurements are summarised in Table 4. The intra-rater RMS-SD of the 3-D CT, 2-D CT and radiographic measurements for the distal tibiofibular syndesmosis were less than 0.94 mm, 0.26 mm and 2.87 mm, respectively. Inter-rater RMS-SD ranged from 0.12 to 1.08 mm in 3-D images, 0.19 to 0.36 mm in 2-D axial images, and 0.47 to 4 mm on radiographs.

**Table 3:** Intra- and inter-rater reliability of CT scan and radiographic measurements

Parameters	Image	Intra-rater		Inter-rater	
		ICC	95 % CI	ICC	95 % CI
XY	3-D CT	0.972	(0.959 to 0.981)	0.966	(0.952 to 0.976)
TCS-A	3-D CT	0.944	(0.918 to 0.962)	0.946	(0.926 to 0.961)
TCS-P	3-D CT	0.953	(0.931 to 0.968)	0.932	(0.900 to 0.954)
TCS-A	2-D CT	0.910	(0.869 to 0.938)	0.902	(0.865 to 0.930)
TCS-P	2-D CT	0.909	(0.869 to 0.938)	0.900	(0.865 to 0.928)
TCS	radiographic	0.838	(0.764 to 0.889)	0.818	(0.754 to 0.869)
TFO	radiographic	0.835	(0.765 to 0.886)	0.762	(0.689 to 0.824)
IFD	3-D CT	0.94	(0.907 to 0.961)	0.907	(0.872 to 0.934)
IFD	2-D CT	0.927	(0.889 to 0.951)	0.895	(0.857 to 0.925)
IFH	3-D CT	0.916	(0.878 to 0.942)	0.908	(0.876 to 0.934)
IFH	radiographic	0.783	(0.694 to 0.849)	0.742	(0.664 to 0.808)

*XY* vertical distance from plane X to plane Y, *TCS-A* anterior tibiofibular clear space, *TCS-P* posterior tibiofibular clear space, *TCS* tibiofibular clear space, *TFO* tibiofibular overlap, *IFD* depth of incisura fibularis, *IFH* height of incisura fibularis

**Table 4:** Intra- and inter-rater precision of CT scan and radiographic measurements

	Intra-rater RMS-SD	Inter-rater RMS-SD		
	Rater 1	Rater 1 vs. 2	Rater 1 vs. 3	Rater 2 vs. 3
3-D CT				
XY	0.48	0.51	0.45	0.61
TCS-A	0.15	0.12	0.14	0.15
TCS-P	0.14	0.17	0.20	0.15
IFD	0.22	0.27	0.20	0.35
IFH	0.94	0.97	0.87	1.08
2-D CT				
TCS-A	0.18	0.19	0.19	0.20
TCS-P	0.24	0.25	0.27	0.20
IFD	0.26	0.27	0.31	0.36
Radiograph				
TCS	0.48	0.50	0.47	0.51
TFO	0.78	0.76	0.76	1.12
IFH	2.87	2.84	2.59	4.00

*RMS-SD* root mean square standard deviation, *XY* vertical distance from plane X to plane Y, *TCS-A* anterior tibiofibular clear space, *TCS-P* posterior tibiofibular clear space, *TCS* tibiofibular clear space, *TFO* tibiofibular overlap, *IFD* depth of incisura fibularis, *IFH* height of incisura fibularis

## Discussion

Syndesmotic injury may be difficult to diagnose using radiographs, because of variability in the amount of rotation, the wide anatomic variability in the depth of the peroneal groove, and the shape of the tibial tubercles. Quantitative parameters of the morphological measurements should be based on a scientific and reliable method; otherwise, the measurement results would not be credible [22]. Accuracy and precision are the most important parameters for evaluation of quantitative measurements for an area of known variability. Traditional common radiographic views for the tibiofibular syndesmosis include weight-bearing AP, mortise, and lateral views of the ankle. Although a mortise view may demonstrate diastasis, it is difficult to perform under post-traumatic conditions. CT is more sensitive than radiography for detecting minor degrees of syndesmotic injury [12], but the selection of the observation plane for 2-D axial CT is still influenced by the position of the ankle. SSD, also known as surface rendering, was the first 3-D rendering technique applied to medical data sets. Its early development in the 1970s was a logical extension of new computer graphic and image-processing techniques and innovations in data segmentation and display [26, 27]. 3-D SSD rendering technology is one of the computer research priorities in the field of visual pattern recognition. Therefore, the 3-D SSD rendering technology was used in our study.

As the syndesmosis plays an important role in stability of the ankle, it was suggested that magnetic resonance imaging (MRI) or CT should be used to ensure adequate diagnosis and evaluation [21, 30]. Franke et al. [31] recommend that persistent dislocation should be verified and detected with intraoperative 3-D imaging or postoperative CT, after reduction and syndesmotic screw fixation of unstable syndesmotic injuries. Malhotra et al. [21] suggested that a comparison of angular and area measurements would help identify a diastasis of the syndesmosis in patients with persistent pain after a rotational ankle fracture. They performed an angular measurement subtended by two lines drawn tangent to the anterior and posterior surfaces of the distal tibia and lateral malleolus. The area measured was bounded by above two lines, the lateral tibia and the medial aspect of the lateral malleolus.

Recently, the lateral view of the ankle has been studied with regard to the relationship of the distal tibia and fibula. Grenier et al. [32] have established a novel radiographic parameter based on the lateral view of the ankle, i.e., the anteroposterior tibiofibular (APTF) ratio, which is normally 0.94 ± 0.13 and can be used to identify and prevent a malreduction of the fibula in the incisura fibularis. Croft et al. [33] showed that the anterior tibiofibular ratio (defined as the ratio of the tibial width to the anterior tibiofibular interval) is reproducibly measured, and is suitable for determining the normal relationship of the tibia and fibula. Some authors used CT to postoperatively evaluate the reduction of the injured syndesmosis [21, 27, 34]. Parameters analogous to those traditionally measured in previous studies [18, 34, 35], including the anterior TCS, posterior TCS and others, were measured using 3-D and 2-D CT. According to these parameters, measurements of the tibiofibular syndesmosis aided in the diagnosis of diastasis. With the increasing use of CT, the rotational changes of the syndesmosis can be measured on axial slices. The rotation of the fibula relative to the tibia is a common parameter; this is described by an angle between a line tangential to the anterior and posterior tibial incisura and a line through the anterior and posterior fibular tubercles [17, 35]. Moreover, Ebinger et al. [36] demonstrated that the rotational displacement of the fibula using a full 3-D CT model, correlated with the average distance between anterior and posterior tibiofibular joint space. In terms of the vertical displacement of the fibula, they indicated that the longitudinal displacement correlated with the posterior tibiofibular joint space measurement on axial CT imaging.

The common measuring plane of the distal tibiofibular syndesmosis on radiograph is 1 cm proximal to the tibial plafond [10, 11, 15, 16]. Elgafy et al. [18] reported that the width of the tibiofibular syndesmosis on the axial CT images was measured in the third section, which was 3 mm thick and 9-12 mm proximal to the tibial plafond. Due to the existence of anatomic variability, a uniform measurement plane lacked a scientific basis. In our study, based on theoretical computational geometry, we precisely located the tibial plafond and the lateral prominent point of the anterior tubercle (point A, defined as the intersection of the tangential along the tibial tubercle) on the 3-D SSD images. The measurement plane through the lateral prominent point and parallel to the tibial plafond was then selected. The maximum TCS or TFO and IFD were considered through the plane, on which relevant parameters were measured. The method could help locate the measuring plane accurately, which differed by sex and anatomic variability in 484 samples. The results showed that the sections were 12.1 mm proximal to the tibial plafond in the male group and 7.8 mm in the female group. The parameters provided comparable measurements planes on the axial CT image and radiographs.

A radiological study using cadavers demonstrated that normally there is a TCS of less than 6 mm on the AP and mortise views; the TFO on the AP view is greater than 6 mm, or 42 % of the fibular width, and greater than 1 mm on the mortise [10]. Ostrum et al. [15] reported a TCS of less than 5.16 mm in females and 6.47 mm in males on AP view radiographs; a TFO more than 2.1 mm in females and 5.7 mm in males indicates an intact syndesmosis. The results for the TCS described in the present study are different from those of previously reported radiographic studies. Elgafy et al. [18] reported that the mean anterior and posterior width of the distal tibiofibular syndesmosis in CT scans, were 2 mm and 5 mm in male, respectively, and 2 mm and 4 mm in women. Yeung et al. [37] demonstrated that axial CT measurements of the tibiofibular distance (TFD) were useful predictors for syndesmosis instability in fractured ankles. They measured the TFD in both normal and fractured ankles. The mean TCS-P measured in the current study was 3.6 mm in the male group and 2.9 mm in the female group. This was comparable to the posterior TFD measured between the analogous reference points on axial CT imaging, which is normally 4.1 mm [37]. In fractures involving the tibia and fibula, 3-D or 2-D measurements could be performed as in normal ankles, when the measurement plane above the tibial plafond is previously well defined. Meanwhile, the pre-defined osseous points are identified regardless of the displacement. In the current study, the TCS was divided into two components on the 3-D SSD and 2-D axial CT images, i.e., anterior and posterior. The two parameters did not differ significantly between the 3-D and 2-D images, because the measurement plane on the 2-D axial CT image was defined precisely by 3-D images. According to the defined TCS, there was a significant difference between posterior TCS in the 3-D image or 2-D axial image and the TCS on the AP view radiographs.

Although TFO was an important measurement for assessment of the syndesmosis on radiographs, it was difficult to assess on the 2-D axial CT section or 3-D image, because the measurement views changed with the rotation of the foot. Therefore, only the TFO on the radiograph was measured in the present study. The accuracy and reliability of TFO would be affected, if it was not measured on a standard AP or coronal radiographic view. The IFD of the tibia may predispose to displacement of the fibula in association with fracture dislocation. Ebraheim et al. [13] found that the depth varied from 1.72 to 6.78 mm with an average of 4.29 mm at the level of 10 mm proximal to the tibial plafond. However, they pointed out that this wide variability in the IFD of the tibia, and in the shape of the tibial tubercle would make evaluation difficult. In our study, we measured the depth in the 3-D images and 2-D axial images using a precisely located plane via the intersection of the tangent along the tubercle and parallel to the tibial plafond. Therefore, we considered the depth value to have a particular significance, which could compensate for anatomic variability.

The limitation of our study was mainly that the software for 3-D morphological evaluation was not commonly available. However, the 3-D SSD rendering technique based on the data of DICOM 3.0 format is accurate; in addition, 3-D measurement technique is already widely used in the field of engineering modeling. Second, the cases included in the study had forefoot injuries or pathology, but not injuries of the tibiofibular syndesmotic ligament complex, according to radiographs and MDCT scans. Third, a CT scan is necessary for the 3-D measurement technique, which causes radiation exposure. However, technological developments have led to a significant reduction in radiation exposure and image noise in the latest MDCT generation [38, 39].

## Conclusions

The current study demonstrates that the measurement plane for normal tibiofibular syndesmosis should not be at a fixed level above the tibial plafond. In particular, the sex difference of the measurement plane should be considered. 3-D measurement technique would help identify the unique and precise measurement plane. Reliable measurements could be provided on that plane, either with 2-D or 3-D MDCT images. This study also provides a series of parameters for the normal tibiofibular syndesmosis for use in diagnosing injuries.

## Additional file

Additional file 1:
**This video showed part of major procedures mentioned in the study as follows: First, three-dimensional images of the ankle were extracted by 3-D interactive and automatic segmentation technique after SSD reconstruction, and the color of labeled bones gradually turned pink (talus), silvery (fibula) and green (tibia).** Second, the measuring plane (called plane Y in the text) was selected individually. The talus was hided and the distal tibia articular surface was clicked to create a pseudo-straighten plane as the tibial plafond (called plane X in the text). “Point A” was selected on the anterior tubercle, which was defined as the intersection of the tangential along the anterior and posterior tubercle. The plane via point A paralleling the tibial plafond (plane X) was described as the measuring plane. The perpendicular distance between two planes was measured. At last, after reference points were conformed, measurements for some parameters were carried out. For example, the posterior tibiofibular clear space (TCS-P) was measured in the 3-D image, as well as in the 2D axial image of CT scans as control. In the 3-D image, the clear space of the posterior tibiofibular at the level of plane Y was captured expediently for further measurement. TCS-P in the 2-D axial images was measured in multiple planar reconstruction (MPR) images at the same level of the measuring plane. (MP4 16470 kb)

## Abbreviations

MD CT: Multi-detector computed tomography
TCS: Tibiofibular clear space
TFO: Tibiofibular overlap
3-D: Three-dimensional
2-D: Two-dimensional
ICC: Intraclass correlation coefficient
RMS-SD: Root mean square standard deviation
AP: Anteroposterior
SSD: Surface shaded display
IFD: Depth of incisura fibularis
IFH: Height of incisura fibularis
CI: Confidence interval
SD: Standard deviation
APTF: Anteroposterior tibiofibular
TFD: Tibiofibular distance
